# Hypnos board: A low-cost all-in-one solution for environment sensor power management, data storage, and task scheduling

**DOI:** 10.1016/j.ohx.2021.e00213

**Published:** 2021-06-23

**Authors:** Bao Nguyen, Bryson Goto, John S. Selker, Chet Udell

**Affiliations:** a Openly Published Environmental Sensing (OPEnS) Lab, OR, USA; b School of Electrical Engineering and Computer Science, Oregon State University, OR, USA; c Department of Biological and Ecological Engineering, Oregon State University, OR, USA

**Keywords:** Open source hardware, Environmental sensing, Low-power state, Data logging, Real-time clock, Relay

## Abstract

Open source in-situ environmental sensor hardware continues to expand across the globe for a variety of applications. Sensor-management systems typically perform three fundamental tasks: sample sensors at a specified time or period, save data onto retrievable media, and switch power to components on and off in between sample cycles to conserve battery energy and increase field operation time. These tasks are commonly accomplished through integrating separate off-the-shelf components into the desired system such as: power relays, SD card hardware, Real-Time Clocks (RTCs), and coin cell batteries. To enable faster prototyping, the Openly Published Environmental Sensing Lab abstracted all of these requirements into a single printed circuit board (PCB), Hypnos, that can be included in any project to achieve these commonly-required capabilities: powering on and off connected sensors on a schedule and logging collected data to the removable SD card. The hardware is laid out in a “Feather” form factor, a popular configuration in the open-source hardware community, to easily mate with other industry standard products. The onboard RTC acts as an alarm clock that wakes a user-attached microprocessor from low-power sleep modes in between sample cycles. By integrating all these components into a single PCB, we save cost while significantly reducing physical system size. The design as well as a suite of code functions that enable the user to configure all the Hypnos board features are detailed.

## Graphical abstract

**Figure:**
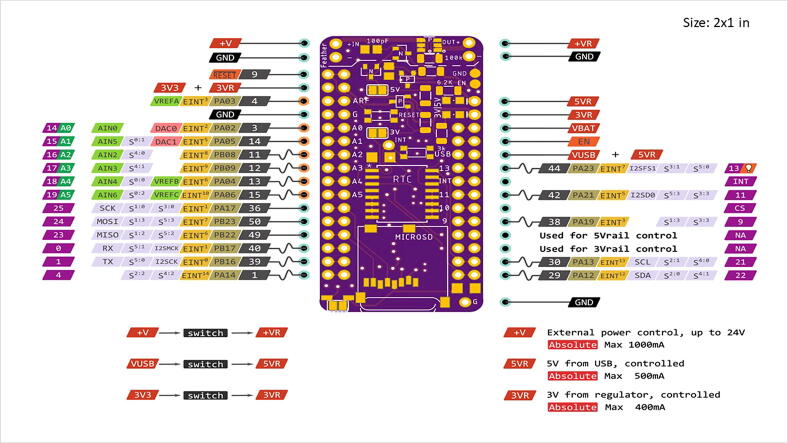

## Specifications table

**Table:** 

Hardware name	*Hypnos Board*
Subject area	Environmental, Planetary and Agricultural Sciences
Hardware type	Electrical engineering and computer science
Open Source License	GNU General Public License v3.0
Cost of Hardware	*Circuit Components: $17.14 + $10.35 (for minimum order of 3 boards)*
Source File Repository	GitHub: https://github.com/OPEnSLab-OSU/OPEnS-Hypnos Zenodo: https://zenodo.org/record/4752671

## Hardware in context

In situ sensor systems translate environmental phenomena at their field site into data that are logged to media (often SD card) to be retrieved for research. Although the variety of what can be measured and for what purposes vary widely, there are certain tasks that are common among a majority of these different projects. Such examples are high-precision time keeping for scheduling sample cycles, saving collected data, as well as powering sensors on and off to conserve power. These tasks are normally done through different hardware-- which commonly require three different pieces: real time clocks (RTCs), SD card memory for data storage, as well as power relays for turning peripherals on and off. While many microprocessors are capable of going into low-power sleep modes to conserve battery, the sensors and other system components may continue to draw current during down-time, wasting battery. Power management in sensor deployment is crucial for creating a long-lasting battery life since the energy resources are limited [1], [2].

A project aimed toward creating a low cost environmental monitoring system is Smart Environmental Monitoring and Analysis Technologies (SEMAT) [3]. The goal of this system is to develop a low cost system for aquatic environments that collected data remotely in real time. Power management was crucial for this project as their project was deployed in remote, aquatic locations and having someone replace the battery frequently was impractical. Over time, the system has developed to the point where the required skills to operate and develop the system have been reduced, increasing the accessibility of the device for a wider range of users. Their system solved power management issues by adding an additional timing circuit using MOSFETs and flip-flops with the timing of the system’s clock.

Another method that is commonly used to conserve power is using a power relay. The OPEnS lab has used Adafruit’s Mini Relay FeatherWing, which was designed with the Feather footprint to allow easy installation to circuits with other Feather boards. The problem with this is it uses a latching mechanism. Since this type of design has moving parts, the stability of the component may not last in the long term. There are other types of power relays that do not use a latching mechanism and instead use MOSFET circuitry such as power controllers from Sparkfun and DFRobot. Using MOSFETs provides more stability to the circuit because they do not rely on moving parts. However, these products typically do not use the Feather footprint design, making the integration more difficult than the FeatherWing Relay for Feather based systems.

The goal when creating the Hypnos board was to be able to preserve power and extend battery life, as well as easily support existing sensor peripherals along with Adafruit’s Feather microcontroller. The Feather can be easily programmed through Arduino, a program that can connect hardware and software. The wide use of Arduino has also developed a variety of documentation and information available online. This key feature is the main reason why many electronic deployments use Arduino [4]. By using the Feather form factor, the Hypnos board can be easily added to the Feather by stacking on top or on the bottom of the Feather, similar to Adafruit’s Latching Relay FeatherWing. With this design, it also reduces the amount of skills required to assemble the electronics. The MOSFET circuitry on the Hypnos also provides stability to the power circuit, helping it last longer in deployment and in the long term. The MOSFETs are used to cut off power to peripherals-- making the system’s sleep current much lower. These features, along with the attached RTC and μSD slot, is what separates the Hypnos board from other products.

The Hypnos board provides all three of these common services (e.g. data logging, power management, and timing for sample wake cycles) in one PCB. The Hypnos high-precision DS3231 RTC is used to track time in between sample cycles as well as queue an interrupt signal as a wake-up alarm for the microprocessor. A 3.3 V coin cell battery may be inserted to keep the RTC powered while the rest of the system is asleep, allowing the RTC to remain powered to send its interrupt signal and keep track of time. A μSD holder is built in so users are able to log their data to a long-term memory and retrieve it when convenient. Power can be turned on and off at the user’s discretion, allowing the user freedom to take measurements as needed. Using the Hypnos board allows users to condense their electronics to one PCB-- reducing the amount of required electronics-- rather than connecting three different types of hardware for the same purpose.

## Hardware description

The Hypnos board is a 2x1 inch PCB with two sets of pin connections, a real time clock, and a μSD chip holder. It is designed based on the Feather pinout specification made by Adafruit [5]. We chose this due to an increasing number of DIY electronics products adopting this pin configuration for ease of integration with other devices using the same footprint. The Hypnos Feather headers is designed to connect a microprocessor board with a Feather footprint. The Hypnos Sensor pinout is designed to be connected to additional sensors. Within the sensor pins, there are three switched power rails that are labeled 3.3 V, 5 V, and an additional OUT + rail that can switch up to 24 V, all of which are controlled by the onboard MOSFETs. These MOSFETs have a smaller footprint than a relay and can be driven with the 3.3 V logic levels of the Adafruit Feather microprocessor. Since there are no moving parts, MOSFETs can last longer than a relay at its rated load. When the microprocessor is powered from a 3.7 V battery, only the 3.3 V power supply will be available. A 5 V USB power supply to the microprocessor will provide power to both the 3.3 V rail and 5 V rail. The third, external power supply input/output is separate from the Feather microprocessor, but shares a common ground with the rest of the system. The RTC and SD are powered through the 3.3 V rail. When power is disconnected from the system, an onboard coin cell battery continues to supply power to the RTC.

The Hypnos combines a DS3231 real-time clock with a μSD slot. Adafruit provides an Adalogger FeatherWing that is similar, but instead features a PCF8523, a less accurate RTC, and a μSD slot. For each least significant bit (LSB), the PCF8523 introduces an offset ranging from 4.069 ppm to 4.34 ppm based on the 32.768 kHz clock [6]. The DS3231 features a temperature-compensated crystal oscillator (TCXO) to make it more accurate for environments with high temperature swings. With these features, the DS3231′s offset ranges between 2 ppm and 3.5 ppm and is therefore more accurate than the PCF8523 [7]. Before Hypnos, to achieve both the DS3231 and μSD slots, users would have to combine two separate modules from SparkFun or two FeatherWings from Adafruit. Using Adafruit’s solution gives the stacking capability to connect the two modules but will require modification to the boards to resolve i2c address conflict between the DS3231 and PCF8523 chips (e.g. you cannot use the PCF8523 and the DS3231 with default settings at the same time without an i2c multiplexer).

The Hypnos board does not have a microprocessor, which is why the Hypnos is a peripheral for a microprocessor board. We use a Feather M0 (SAMD12) board in this particular application. The Hypnos provides power switching for the sensors and a medium for the SD card for data logging. The Feather M0 microprocessor turns the Hypnos’ power rails on and off via GPIO, sets the Hypnos DS3231 alarms via i2c, communicates with the sensors, and logs the data to the Hypnos’ SD card through SPI communication. The Hypnos DS3231 interrupt pin is attached to a GPIO pin on the Feather M0 to wake the microprocessor from sleep mode. The Feather M0 has built-in i2c, SPI, and GPIO pins that are designed to support communication protocols to attached sensors and the Hypnos peripherals. Many other microprocessors meet these requirements to use the Hypnos as well. Adafruit has a series of Feathers that accomplish additional tasks such as LoRa communication, WiFi connection, Bluetooth, and more that can be found on Adafruit’s website. If telemetry is desired, the user would attach the appropriate Feather hardware to the system and program it accordingly.

Because the Hypnos does not sample the sensors themselves (it is only responsible for saving data to μSD, interrupting the microprocessor from sleep, and switching sensor power on and off), the range of sensors that can be supported by the Hypnos is limited by the attached microcontroller. The Hypnos i2c line also includes optional pull-up resistors that may increase performance since the Feather M0 does not come with attached resistors [8], [9].

Serial bus hanging, memory overflow, and hard faults can cause embedded systems to crash. For example, a commonly used Wire library to interface with i2c devices with Arduino has no time-out functions and possible infinite while loop conditions. The Hypnos itself does not contain failsafe mechanisms in the case of an i2c or SPI bus crash during operation or sleep. However solutions to these kinds of issues are well known and documented. The Feather M0 (SAMD21), as well as many other microprocessors contain software watchdog timers that can be configured to reset and restore the system in these circumstances [10]. Our lab uses the FeatherFault library for the Feather M0 (SAMD21) which supports detections for hanging, memory overflow, and hard faults as well as reporting and logging crash locations and error codes [11]. FeatherFault macros can be placed in the user’s arduino code that function as waypoints. The most recent waypoint location is logged to a file in SD memory to provide a rough indicator of where in the code a crash might have happened.

The Hypnos has the following overall capabilities:

● Low power control circuit designed for sensor package.
● Combination of high-precision real-time clock DS3231 with μSD slot for data logging.
● FeatherWing footprint for stackable modules.

## Design files

### Design files summary

**Table:** 

Design file name	File type	Open source license	Location of the file
Hypnos V3.2.brd	BRD	GPL 3.0	https://doi.org/10.5281/zenodo.4752671
Hypnos V3.2.sch	SCH	GPL 3.0	https://doi.org/10.5281/zenodo.4752671
Hypnos V3.2_Gerber.zip	ZIP	GPL 3.0	https://doi.org/10.5281/zenodo.4752671
Hypnos V3.2_centroid.csv	CSV	GPL 3.0	https://doi.org/10.5281/zenodo.4752671

**Hypnos V3.2.brd:** The file that can be loaded into AutoDesk EAGLE to get the layout of the board.

**Hypnos V3.2.sch:** The file that can be loaded into AutoDesk EAGLE to get the schematic of the circuit.

**Hypnos V3.2_Gerber.zip:** The Hypnos PCB manufacturing files that can be loaded into Gerblook for free viewing of the Hypnos board [12].

**Hypnos V3.2_centroid.csv:** Pick-n-place file to send for PCB assembly services.

## Bill of materials

**Table:** 

Designator	Component	Number	Cost per unit - USD	Total cost - USD	Source of materials	Material type
PMOS1, PMOS2	MOSFET P-Ch 30 V 3.8A	2	$0.33	$0.66	Mouser	Semiconductor
PMOS3	MOSFET P-Ch 30 V 4.3A	1	$0.39*	$0.39	Mouser	Semiconductor
Q1, Q2	MOSFET N-Ch 60 V 310 mA	2	$0.16	$0.32	Mouser	Semiconductor
R4, R11	100kΩ ± 1% 0.25 W Chip Resistor	2	$0.10	$0.20	Mouser	Non-specific
R1, R2	30kΩ ± 5% 0.25 W Chip Resistor	2	$0.10	$0.20	Mouser	Non-specific
MICROSD	MicroSD Card Socket	1	$1.95	$1.95	Mouser	Non-specific
RTC	Real Time Clock (RTC) IC Clock/Calendar i2c	1	$9.04*	$9.04	Mouser	Non-specific
U$1, U$2	Tactile Switch 50 mA 32 V	2	$0.34	$0.68	Mouser	Non-specific
B1	Coin Cell Battery Holder, 12.0 mm	1	$0.91	$0.91	Mouser	Non-specific
R6, R7	10kΩ ± 5% 0.25 W Chip Resistor	2	$0.15	$0.15	Mouser	Non-specific
C1, C3	0.1µF ± 10% 100 V Ceramic Capacitor	2	$0.26*	$0.52	Mouser	Ceramic
LED1, LED2	Red 625 nm LED - Discrete 1.8 V	2	$0.50*	$1.00	Mouser	Non-specific
R9	3kΩ ± 1% 0.25 W Chip Resistor	1	$0.10	$0.10	Mouser	Non-specific
R8	6.2kΩ ± 1% 0.25 W Chip Resistor	1	$0.23	$0.23	Mouser	Non-specific
C2, C4	100pF ± 5% 50 V Ceramic Capacitor	2	$0.10	$0.20	Mouser	Ceramic
D1	Diode Schottky 20 V 1A	1	$0.44	$0.44	Mouser	Non-specific

Pricing Notes:

* price will vary with source

## Build instructions

### Materials needed

The hardware materials that are needed to assemble the Hypnos board are listed in the Bill of Materials in Section “Bill of Materials**”**. Additionally, there are other tools that are needed to build the Hypnos board such as a soldering iron, lead-free solder, solder paste, flux, a printed stencil of the Hypnos (optional), as well as a reflow oven or heat gun. Alternatively, if a reflow oven is not available, a toaster oven can be used in its place. The printed stencils are available through the CAD files and are used to apply solder paste accurately on the Hypnos PCB. A Hypnos Build Guide video is available for visual instructions.

### PCB assembly

**Fig. 1:**
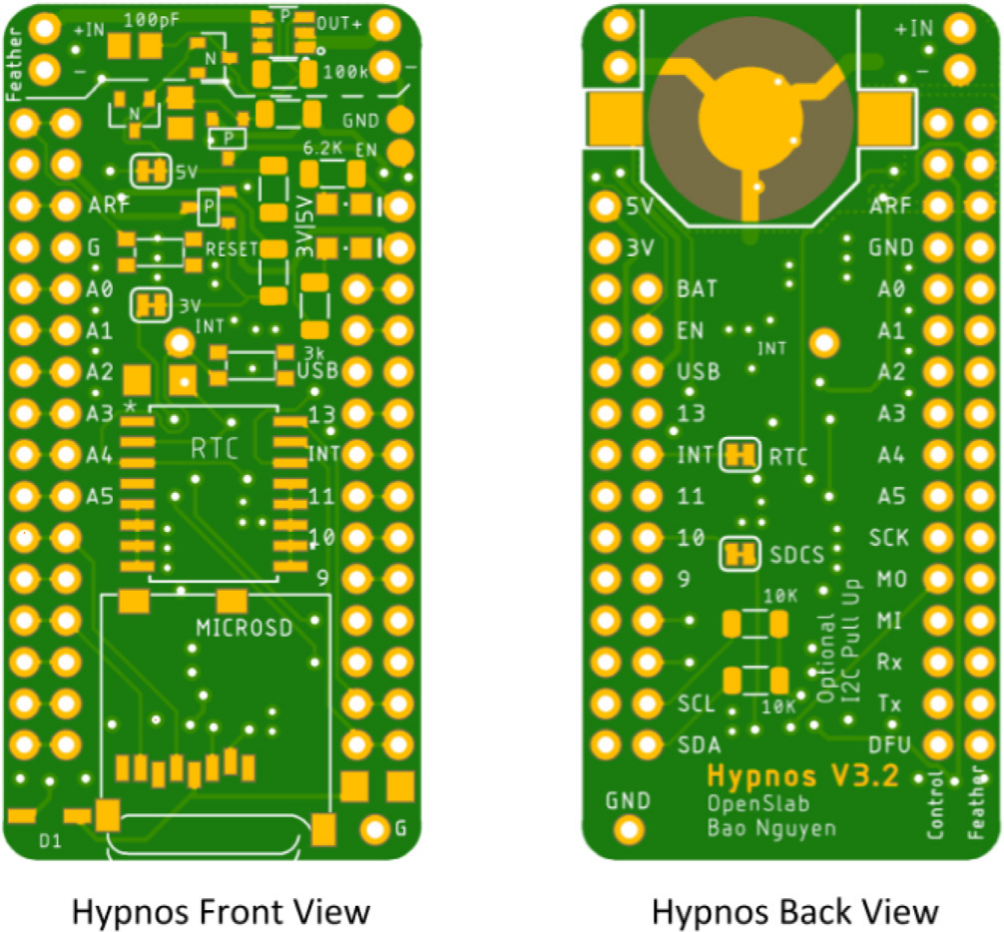
Front and Back View of Hypnos Board.

Assembling the Hypnos will use the reflow oven for the top components but will require hand soldering for the back components. If a reflow oven is not available, hand soldering and reflowing with a heat gun is another option which will be explained in Section“Reflow process”. The layout for the Hypnos board is shown below in Fig. 1. In total, there are 22 components that should be placed on the front of the Hypnos board and 3 components that are soldered to the back-- which is explained in Section “Hand soldering”.

### Apply solder paste

Using the printed stencil, align the Hypnos underneath ensuring that the holes of the stencil line up with the solder pads of the Hypnos. Solder paste will only be applied to the front face of the Hypnos. Apply a small amount of solder paste to each hole-- making sure that the solder paste is on the solder pads and does not smear when the stencil is removed.

### Placing components

**Fig. 2:**
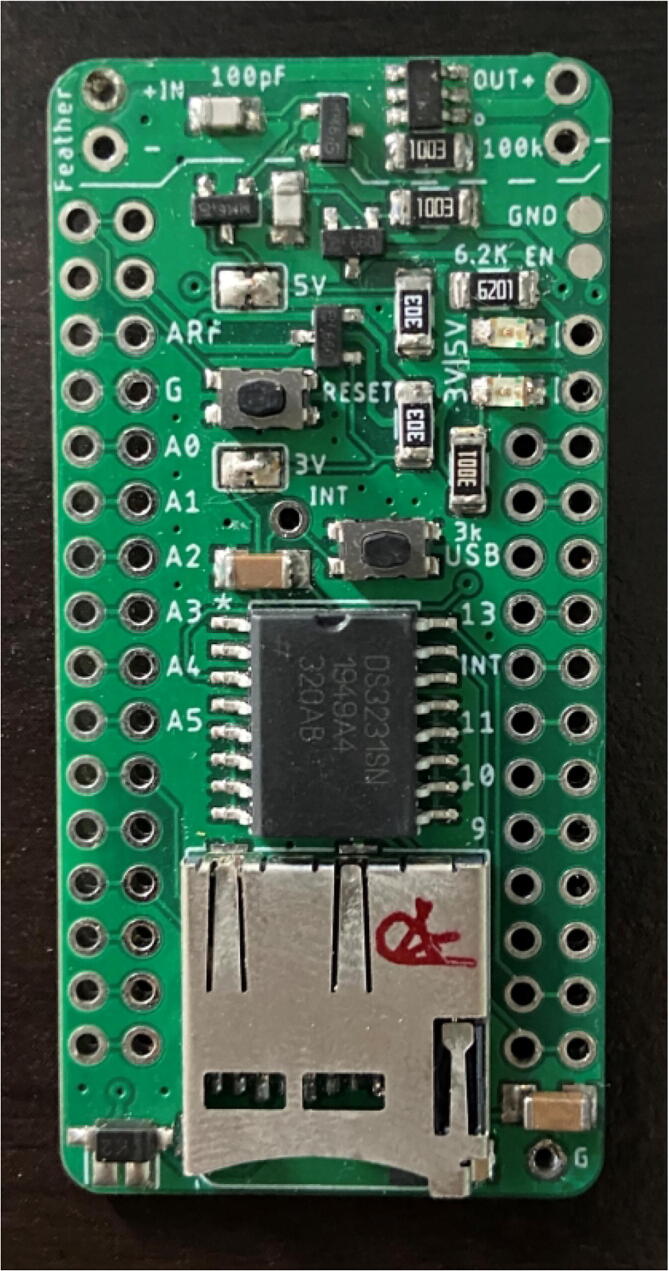
Assembled Top View of Hypnos Board.

There are two options for determining the placement of each component: looking at a picture of a finished Hypnos board (shown in Fig. 2 below) or opening the .brd file with Eagle CAD and placing the components accordingly. There is also a free online viewer that the Hypnos gerber files can be uploaded to view on a browser called Gerblook. Only the front components should be placed for now, the back components will be added after the reflow process.

#### μSD card module

**Fig. 3:**
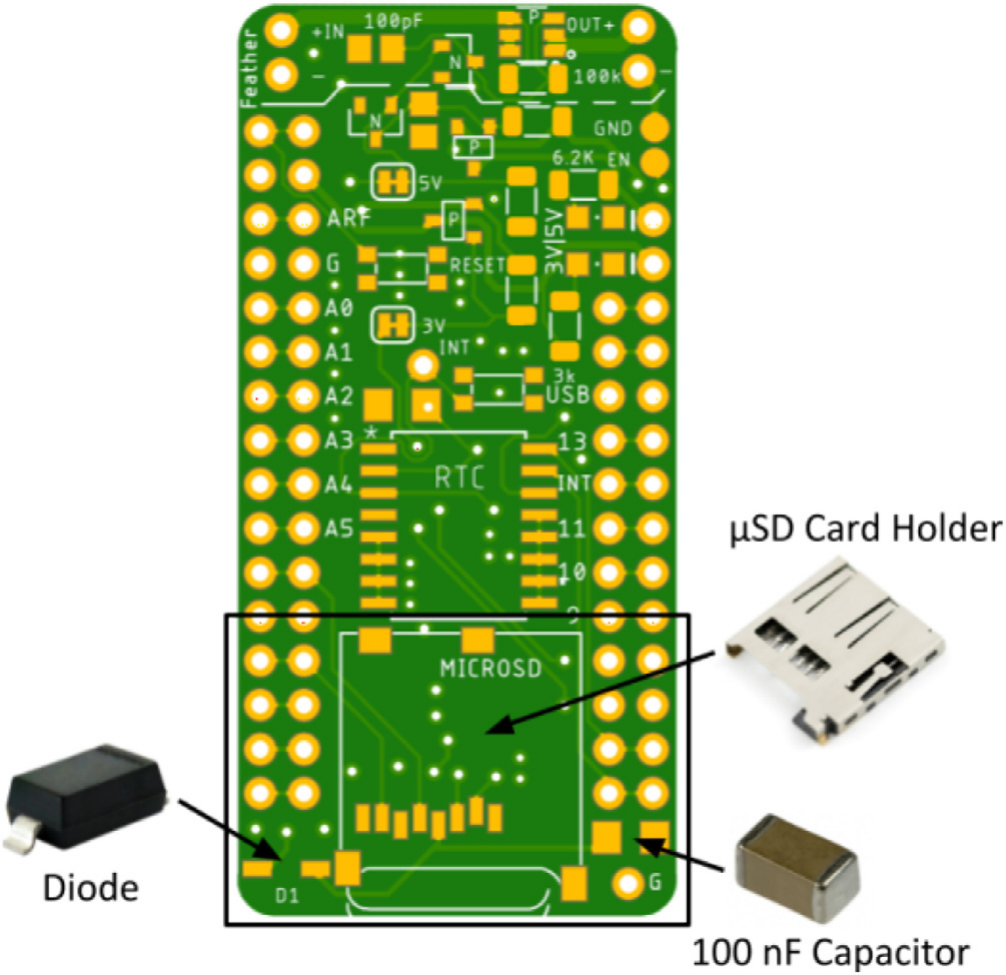
μSD card module: μSD card holder, diode, and 100 nF capacitor placement. Diode polarity indicator facing toward μSD card holder.

The μSD card module has three main components: a diode, a 100 nF capacitor, and the μSD card holder shown in Fig. 3. Place the components ensuring the metal ends of each component are touching the pads on the Hypnos board. Placing the μSD card holder requires extra precision because the pins on the inside of the holder need to be placed so they are touching the pads on the inside. The diode has lines that indicate the direction of the diode. Place the diode so the lines are towards the inside of the board (closest to the μSD holder).

#### RTC with interrupt push button

**Fig. 4:**
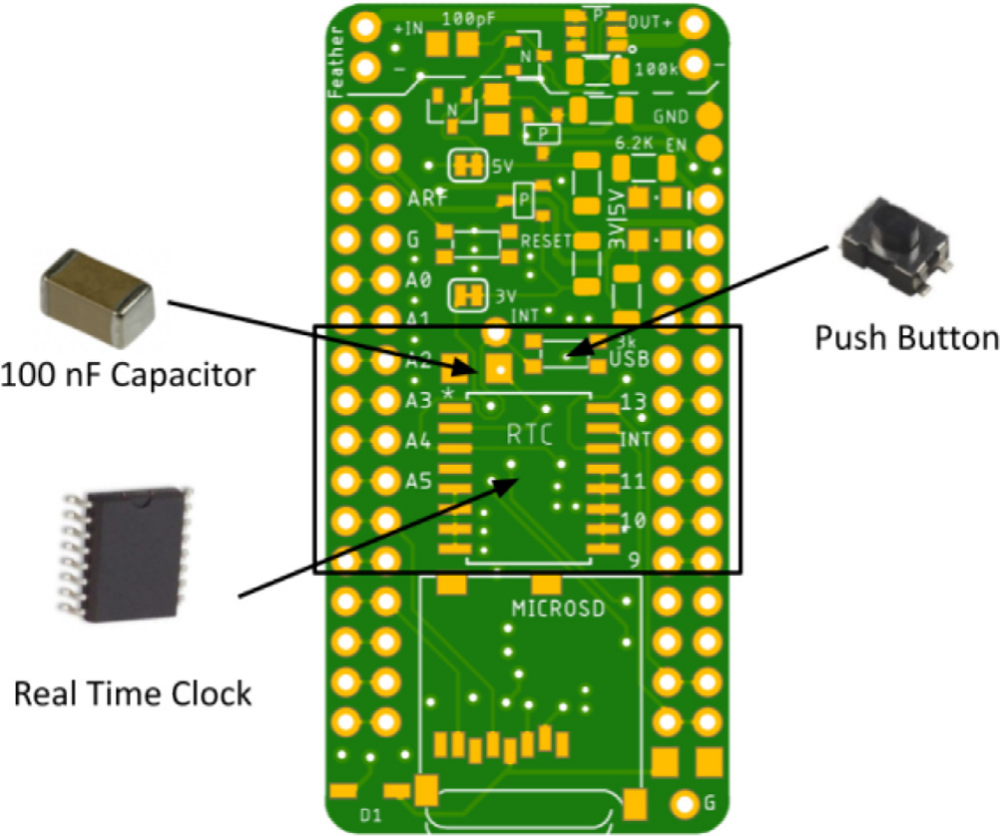
Real time clock, 100nF capacitor, and interrupt push button placement. Polarity notch indicator on real time clock facing toward top of the Hypnos board.

The RTC module contains the RTC, a 100 nF capacitor, as well as a push button that sends an interrupt signal. Again, place the components so the metal ends are touching the pads of the Hypnos board. The RTC is designed to have a notch at the top of the module. Place the RTC so the notch is on the top side. Also be careful to place the RTC so each of the pins are touching only one pad. The orientation of the capacitor and the push button does not matter as long as they are touching the pads. Since these components are rectangular, there are only two configurations where they would touch the pads. The way they are oriented from there does not matter. Fig. 4 shows the appropriate placements.

#### Remaining components

**Fig. 5:**
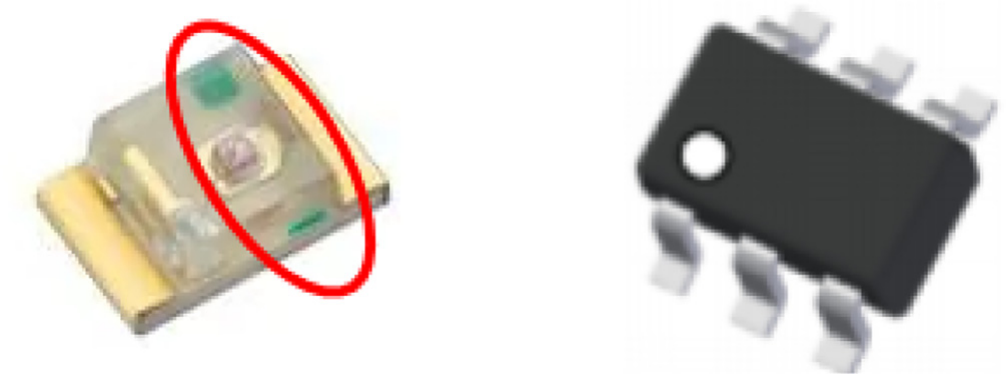
Polarity Indications (Green lining on LED and circle in corner for large P-Channel MOSFET). (For interpretation of the references to colour in this figure legend, the reader is referred to the web version of this article.)

While most of the components do not have a polarity, there are certain components that do, such as the LED and the large P-channel MOSFET shown in Fig. 5. These components were designed to have indications on the polarity of these modules. The LED has a green lining on the inside while the large P-channel MOSFET has a circle in one of the corners.

**Fig. 6:**
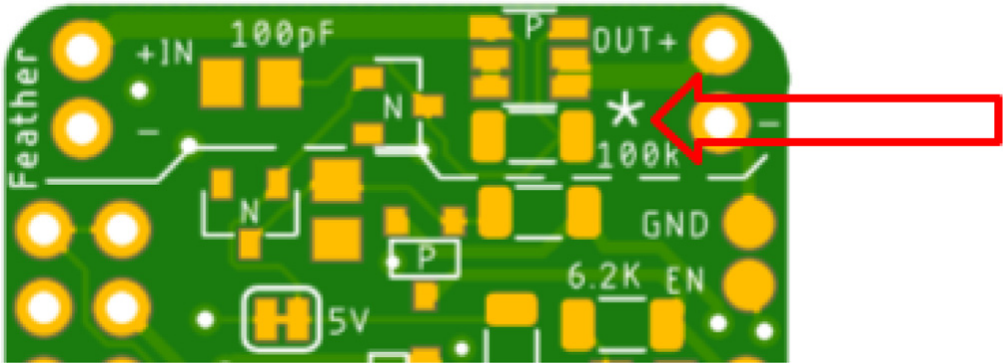
MOSFET Polarity Indicator on Hypnos board.

**Fig. 7:**
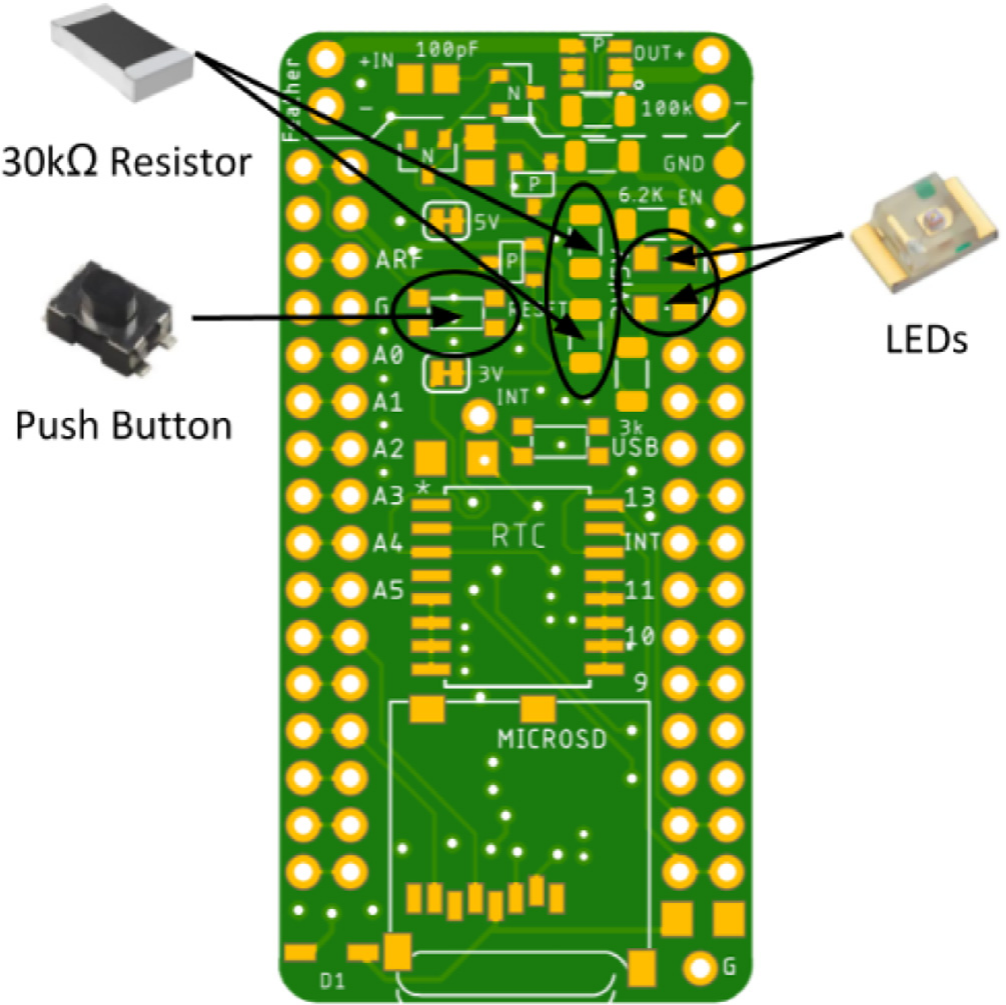
Reset push button, 30kΩ resistors, and LED placement. Green lining polarity indicator facing outward for LED. (For interpretation of the references to colour in this figure legend, the reader is referred to the web version of this article.)

**Fig. 8:**
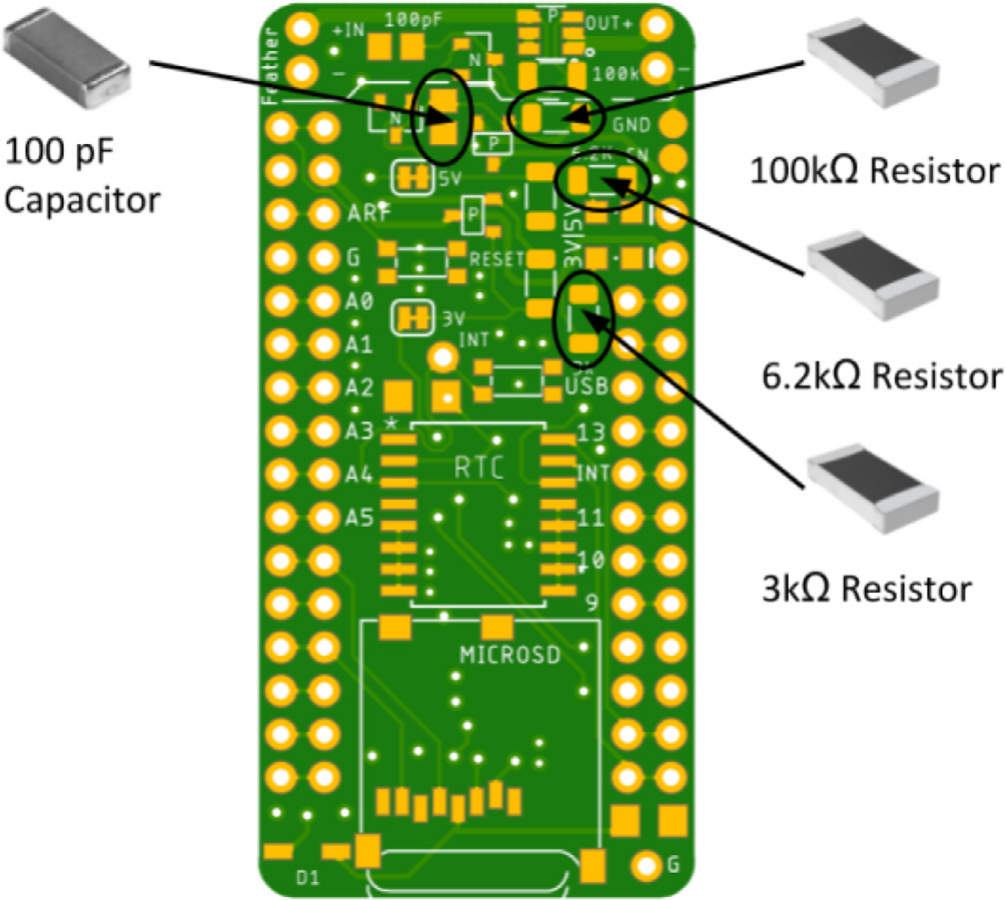
100kΩ, 6.2kΩ, and 3kΩ Resistor and 100 pF Capacitor Placement.

**Fig. 9:**
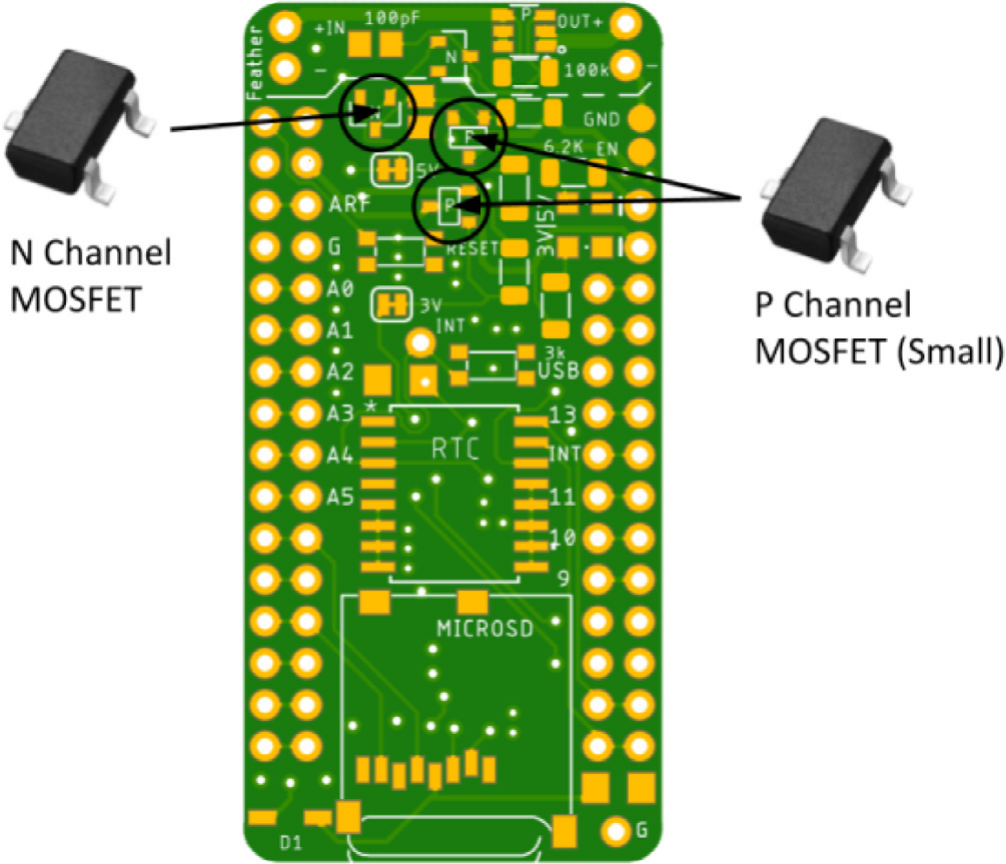
MOSFET Circuitry Placement.

**Fig. 10:**
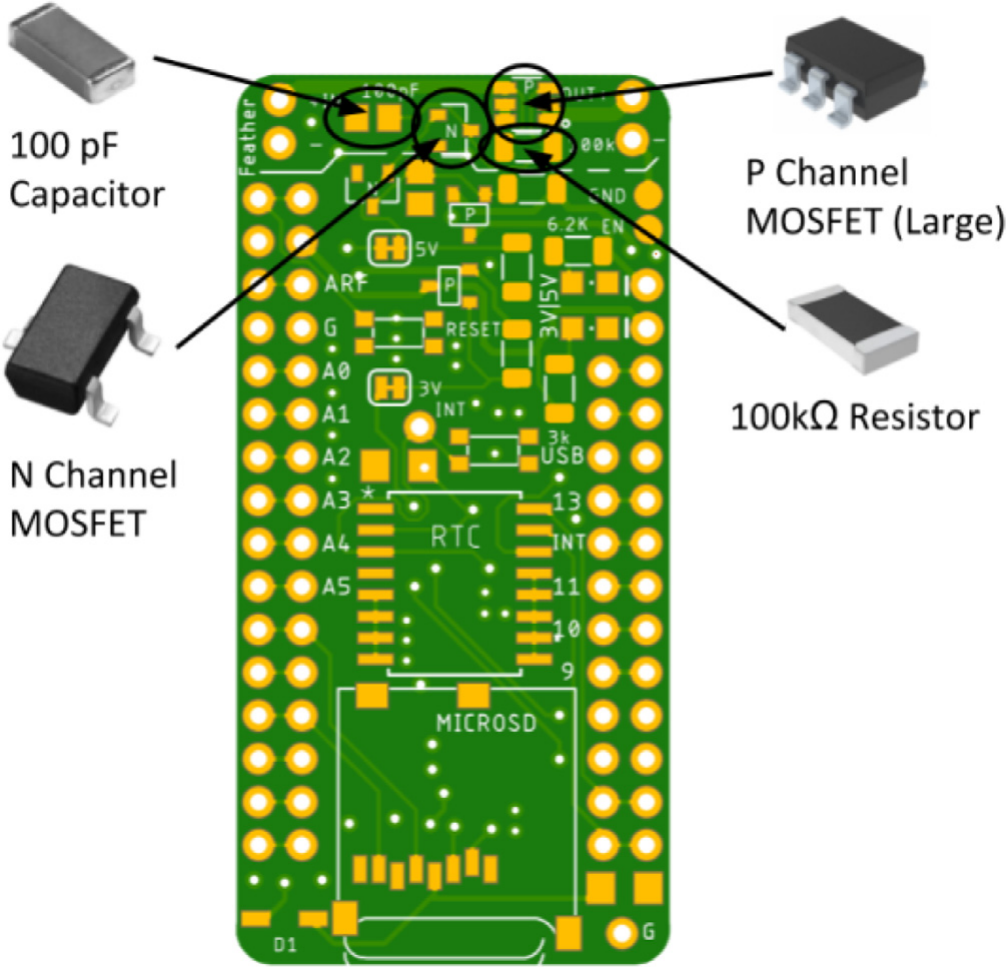
+ V Rail Component Placement: Match circled polarity indicator of large P-Channel MOSFET with Hypnos’ circled polarity indicator.

Place the LED so the green lining is facing the outside of the board. The large P-channel MOSFET should be placed so the circle is in the bottom right corner. The Hypnos board has an asterisk that indicates this shown in Fig. 6. The placement of the remaining components are shown in Fig. 7, Fig. 8, Fig. 9, Fig. 10 below. Once all of these components are placed, the board is ready to be reflowed.

### Reflow process

**Fig. 11:**
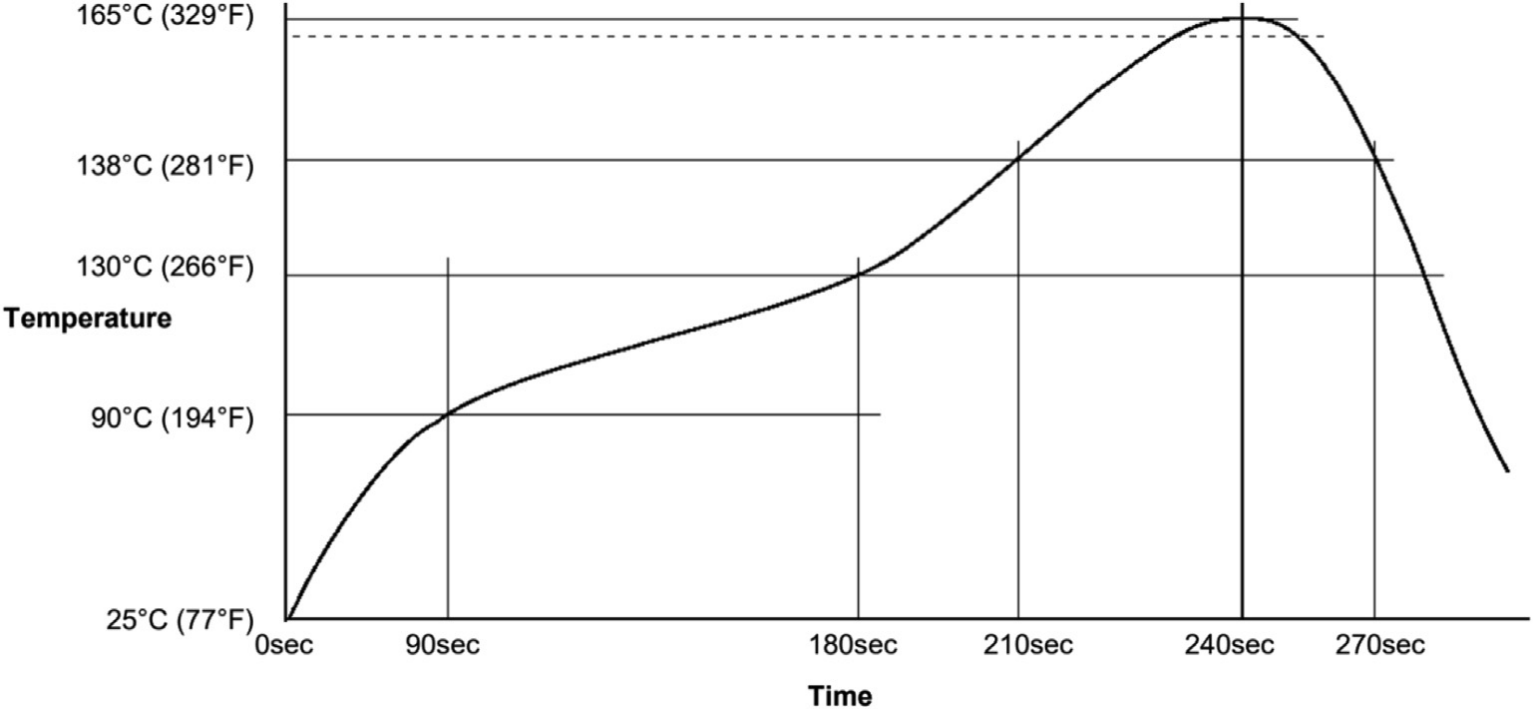
Low Temperature Solder Reflow Profile: Sn42/Bi57.6/Ag0.4 solder assembly, designed as a starting point for process optimization [13]

After placing the components on the Hypnos board, place the PCB into the reflow oven and heat it up according to the low-temperature solder reflow graph in Fig. 11. Be sure to follow proper safety procedures when doing the reflow process as it may cause injuries and burns. The OPEnS lab has created a guide to the reflow process with either the reflow oven or a standard toaster oven. Although the RTC is temperature sensitive, it can handle temperatures below 180 °C.

If a reflow or toaster oven is not available, the top components can also be hand soldered with the addition of a heat gun. It is recommended that components are soldered one at a time starting with the μSD holder since it has many pins that may be difficult to solder with a soldering iron. Apply solder onto the pads that the μSD holder connects to first. Then, place the μSD holder and use the heat gun to heat up the solder to create a connection. Once the board is cooled, the remaining components can be hand soldered one at a time with a similar technique of applying solder to the pads first and then placing the components. The soldering iron can be used to heat up the solder instead of the heat gun for the rest of the process.

### Hand soldering

**Fig. 12:**
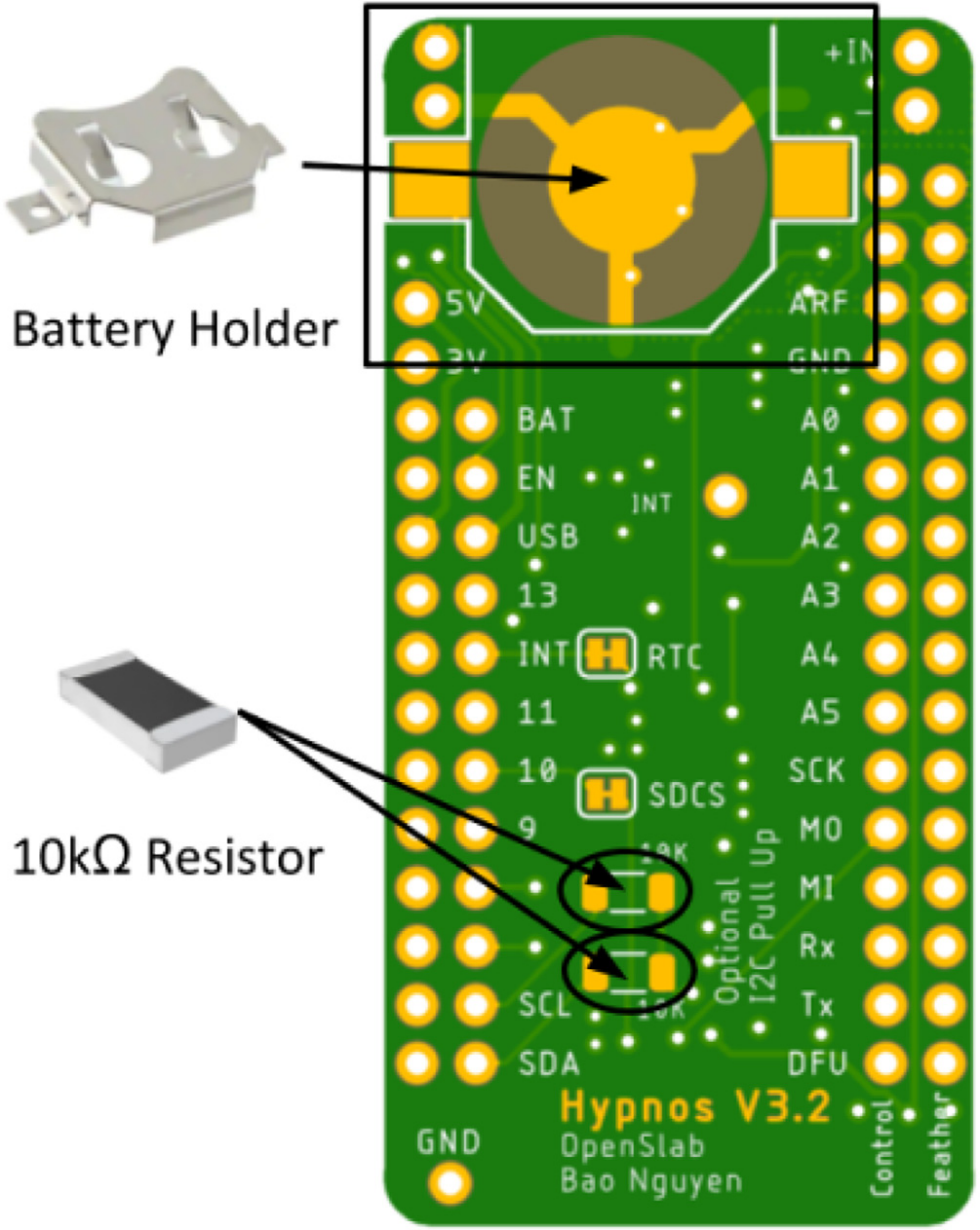
Hand Soldered Component Placements.

Once the reflow process is complete and the Hypnos board is cooled, hand solder the remaining components to the back side as shown in Fig. 12. There are three components that should be soldered at this stage: two 10kΩ resistors and the battery holder. Extra solder is required for the battery holder since it tends to come off easily with light-solder. The orientation of the resistors does not matter, but the battery holder should be soldered so the open slot is facing outward. This will allow easy access to the coin cell battery.

### Solder headers onto Adafruit Feather M0 and Hypnos

**Fig. 13:**
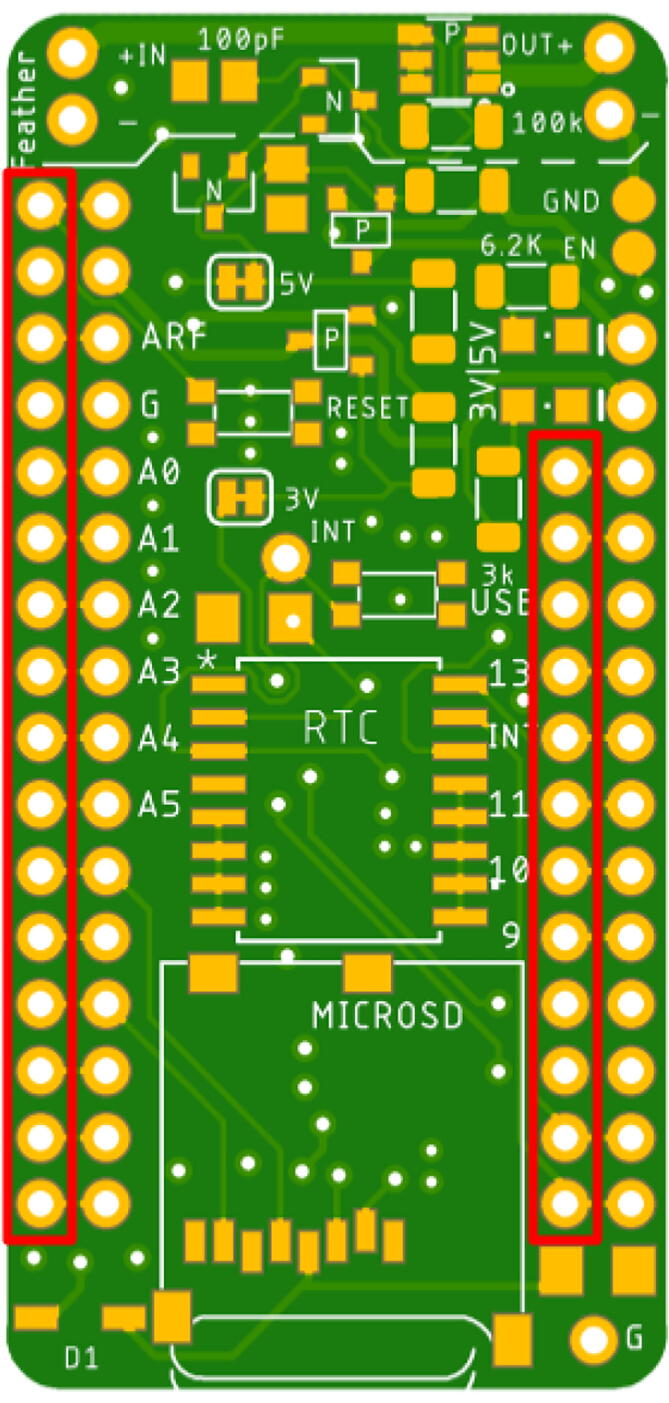
The Feather needs to connect to these header rows (red). (For interpretation of the references to colour in this figure legend, the reader is referred to the web version of this article.)

**Fig. 14:**
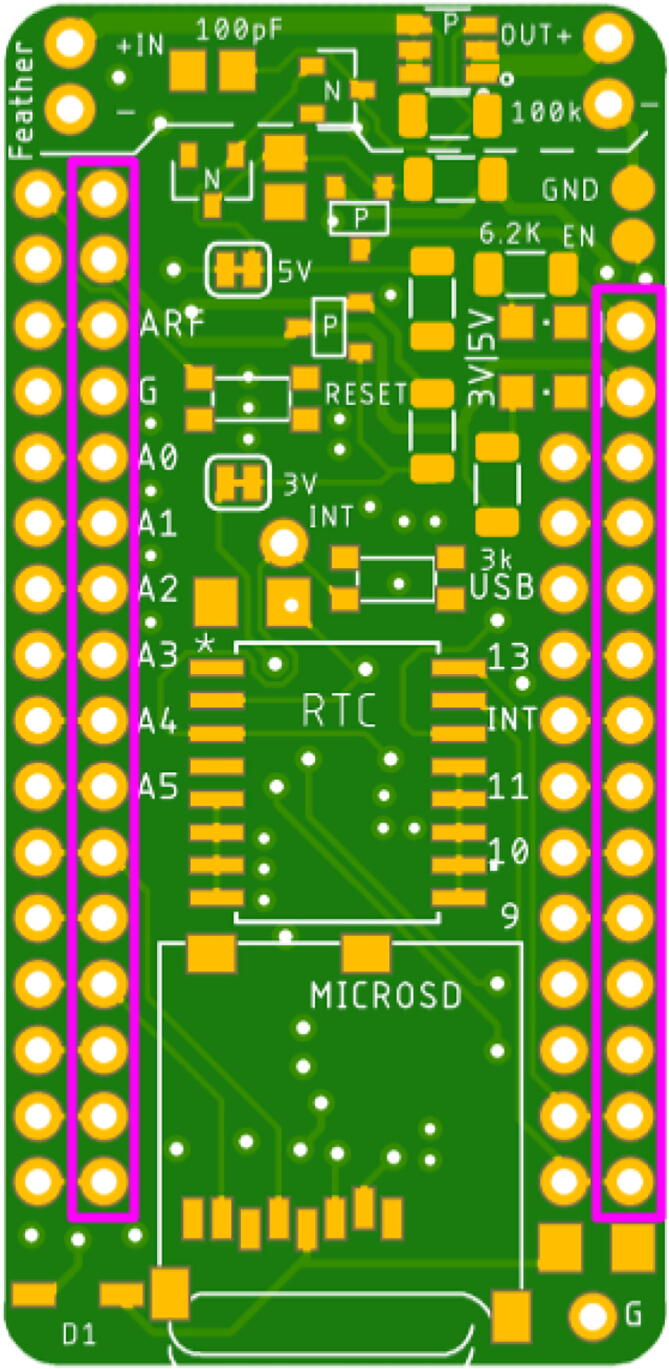
Sensors or sensor expansion board connect to these header rows (pink). (For interpretation of the references to colour in this figure legend, the reader is referred to the web version of this article.)

**Fig. 15:**
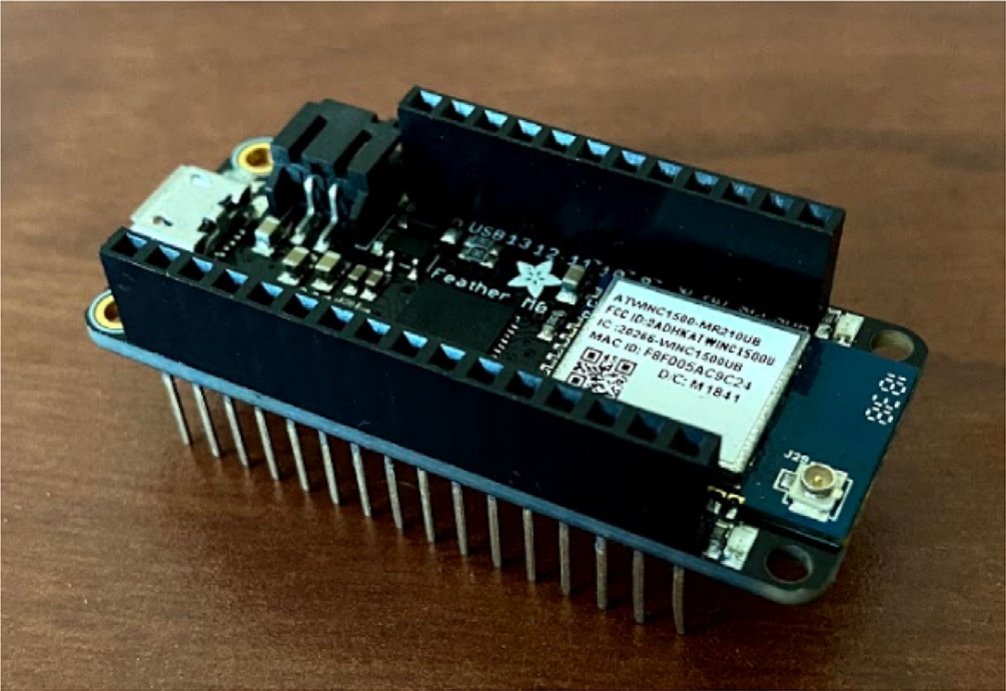
Soldered stacking pins on Feather.

Solder desired headers to connect to a Feather M0 board (both on Hypnos and Feather). When deciding which headers to solder on, it is important to think about how the Feather and Hypnos will connect to the sensor or sensor board. Note that the power rails on the Hypnos are shown in Fig. 13, Fig. 14 as well as an example picture of a Feather with headers in Fig. 15. There are three main ways to connect the Hypnos in relation to the Feather and the sensor / sensor board: 1) stacking the Hypnos between the Feather and the sensor board, 2) using a FeatherWing Doubler or Tripler to connect these boards, or 3) stacking the Feather and Hypnos and directly wiring the sensor to the Hypnos. Based on the sensor expansion board mechanical layout, one has to decide to place the Feather micro-controller board on top or bottom of the Hypnos. More details about this are covered in the next sections.

### Connect Feather board to Feather pinout on the Hypnos board

**Fig. 16:**
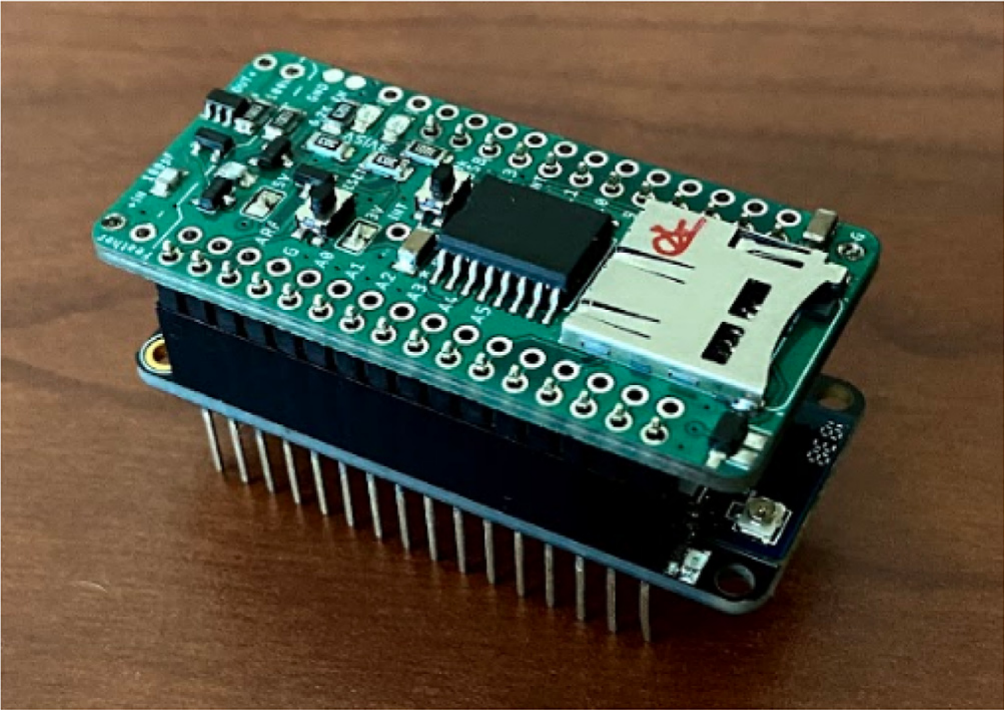
Hypnos stacked on top of Feather.

The Feather will stack on top (or on the bottom) of the Hypnos. As Fig. 16 shows, the Feather is stacked on the Feather pinout shown in Fig. 13. There are a few considerations to think about when determining whether to connect the Feather on top or on the bottom of the Hypnos. The first is the accessibility to the Hypnos and its components. The Hypnos comes with the μSD as well as the coin cell battery which are placed on the top and bottom of the Hypnos respectively. Another consideration is having access to the push buttons. The Hypnos comes with a push button for resets and interrupts. If a project would need access to these buttons frequently, having the Hypnos placed on top would be more convenient.

### Connect sensor and/or sensor board

As mentioned before in Section “Solder headers onto Adafruit feather M0 and hypnos”, there are 3 main ways to connect the Hypnos in relation to the Feather and sensor/sensor board: stacking the Hypnos between the Feather and sensor board, using a Feathering Doubler or Tripler to connect all the electronics, or stacking the Feather and Hypnos and wiring the sensor to the Hypnos rails.

**Fig. 17:**
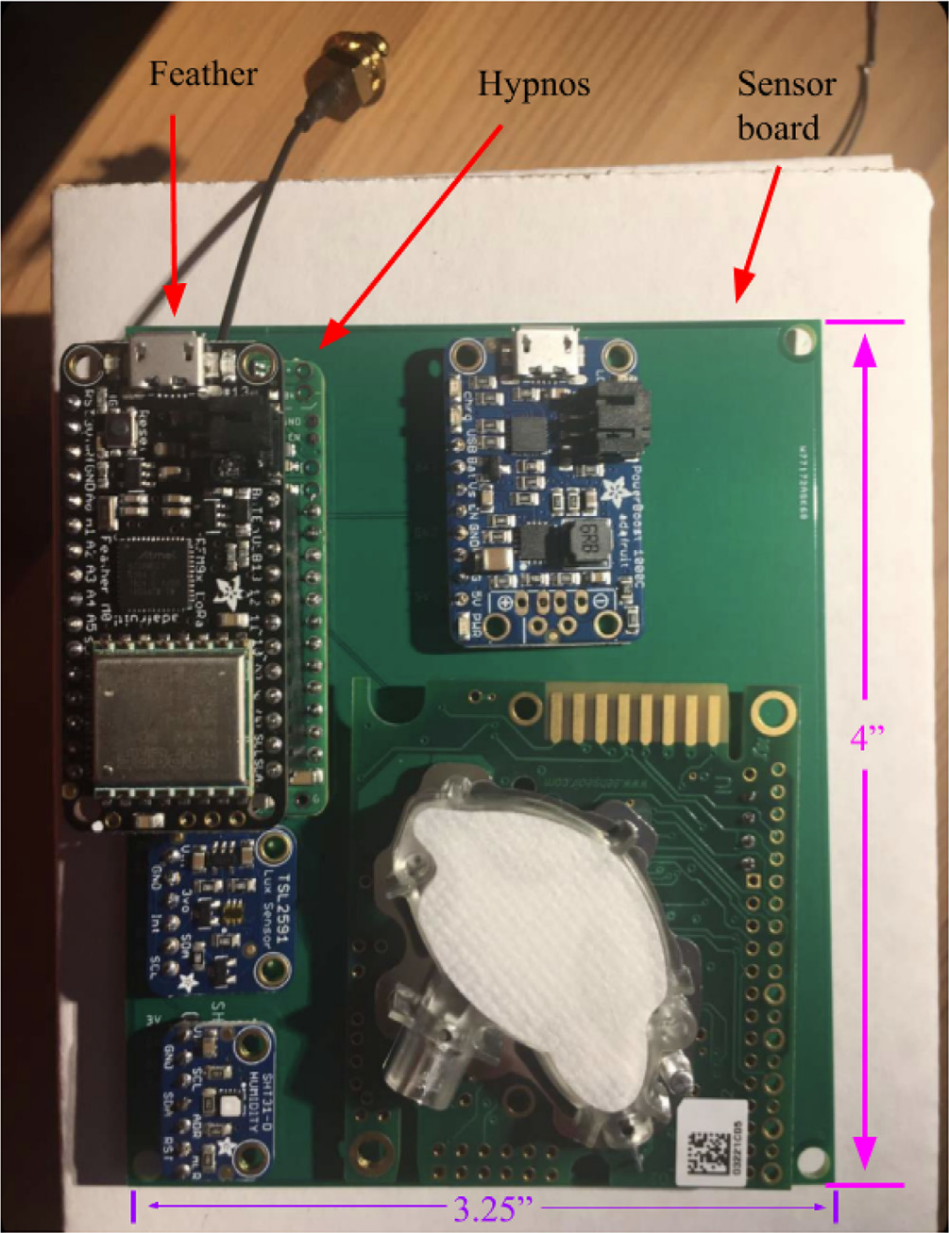
Hypnos stacked between Feather and sensor board.

Fig. 17 below shows an example of an electronic setup where the Hypnos is stacked in between the Feather and sensor board. The following configuration was decided because the sensor board had multiple sensors on top and because the sensor board was the largest PCB. In this setup, the Feather had male headers soldered onto it to connect to the Hypnos. The Hypnos board had female headers soldered onto the Feather pinout and male headers soldered onto the sensor pinout. This header configuration allows for the Hypnos to attach to the Feather and sensor board nicely.

**Fig. 18:**
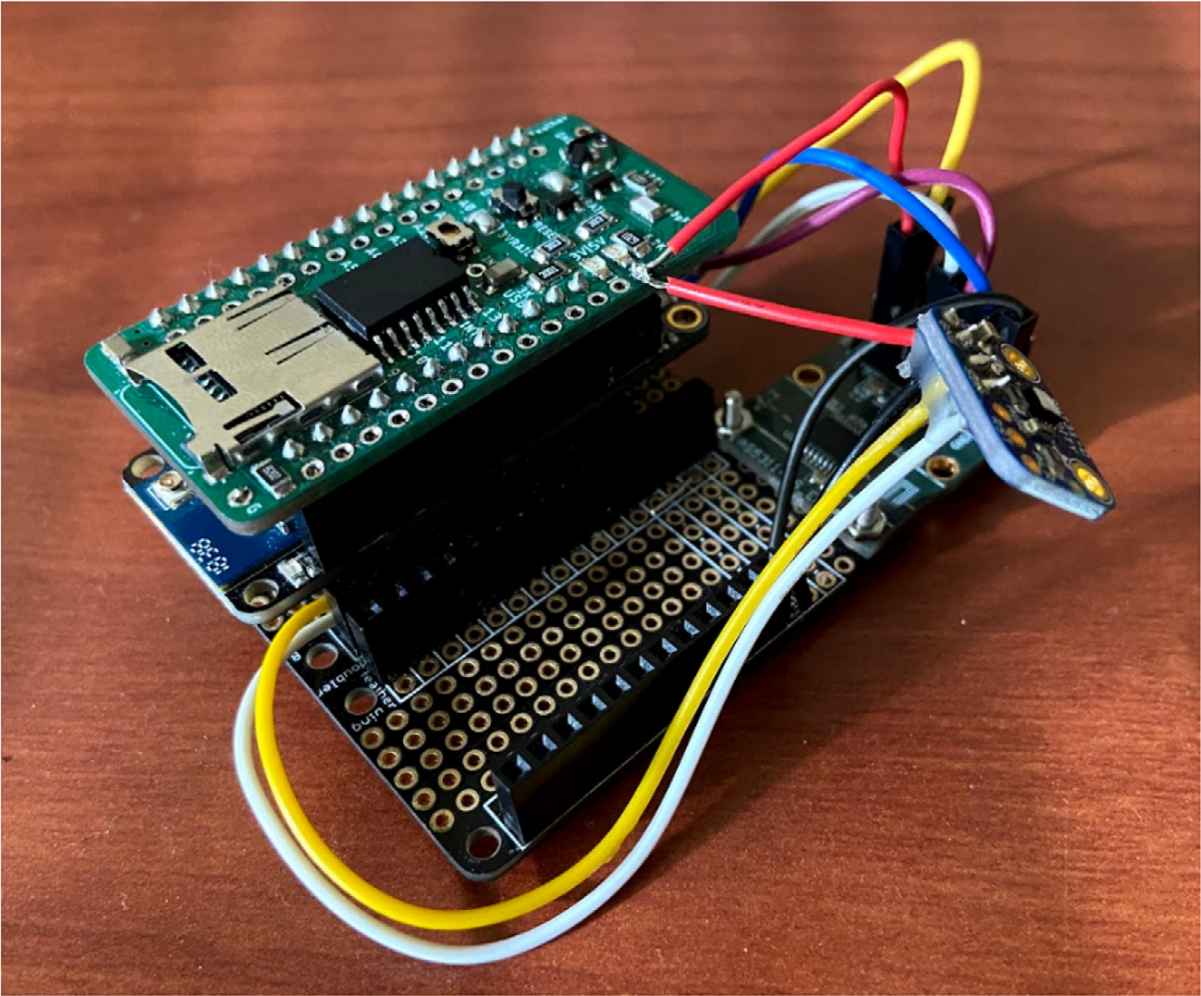
Hypnos stacked on top of the Feather and connected to external sensors.

The second configuration is shown in Fig. 18 below where the FeatherWing Doubler is used to connect the Feather, Hypnos, and other sensors. This setup was chosen because the sensors were not able to stack on the Hypnos. Thus, the Doubler was picked to keep the electronics together and make wiring two sensors easier.

**Fig. 19:**
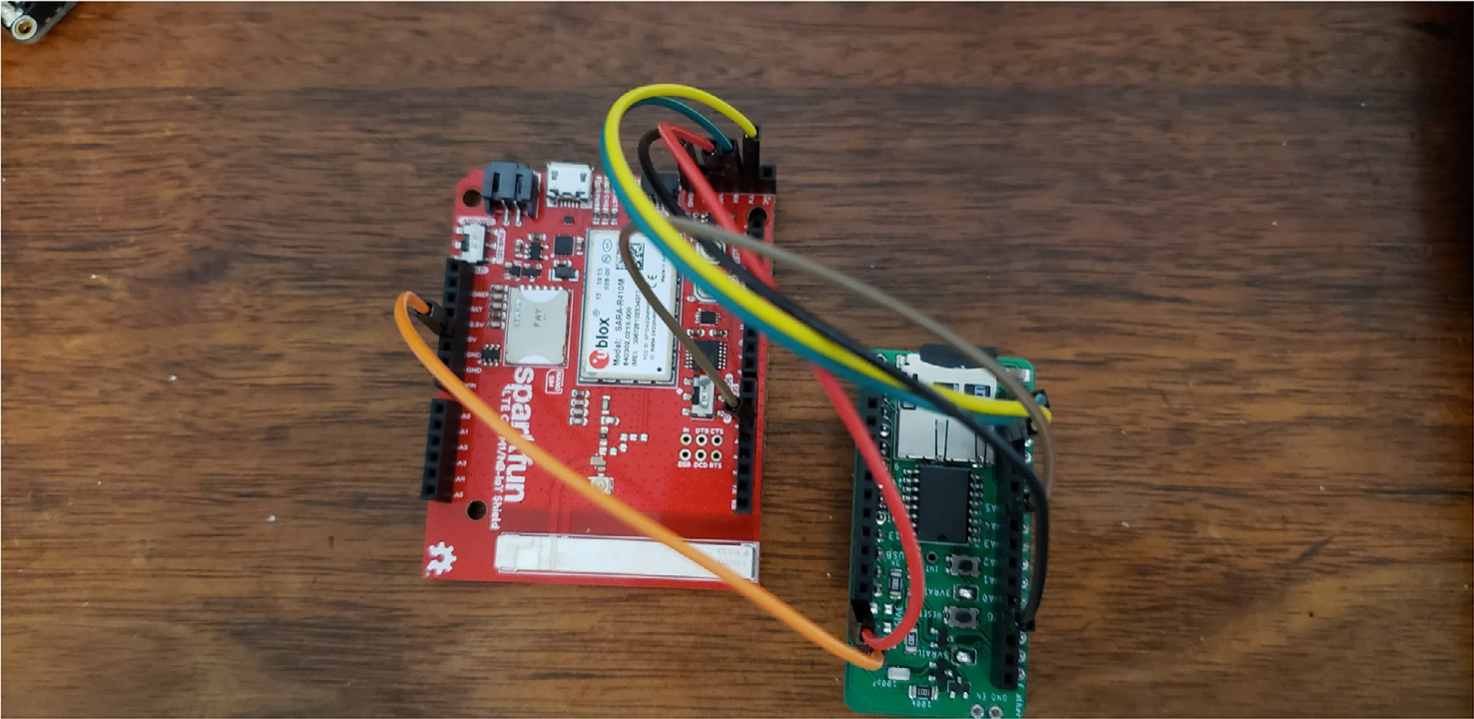
Hypnos stacked on top of Feather and manually wired to sensor.

The third configuration is stacking the Hypnos on top of a Feather and wiring the sensor wires to the Hypnos as shown in Fig. 19. Since this particular sensor did not require many wires, the sensor was wired to the Hypnos to simplify the setup process. This setup is good if an external PCB or FeatherWing Doubler/Tripler is not available or if rapid prototyping is convenient.

In all of these examples, the Hypnos was placed depending on the needs of the project. In the first example, the Hypnos was placed between the Feather and sensor board. Placing the Hypnos between the two will prevent access to the two push buttons, but it was required for the circuit to work. The second and third example had the Hypnos stacked on top of the Feather which allowed the sensor wires to be easily hooked up to the power rails on the Hypnos. This configuration also allows access to the push button in the event the reset and interrupt buttons are needed.

### Solder sensor wires to sensor pinout on Hypnos

**Fig. 20:**
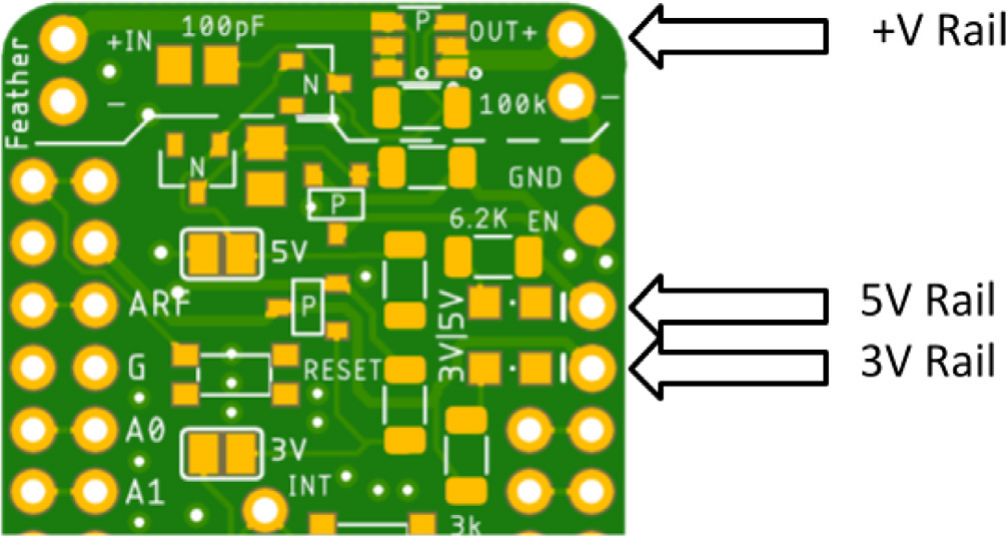
Voltage Rail Diagram.

Wiring the sensor wires to the Hypnos will be similar to wiring them through a Feather with the only difference being where the sensor’s power wire is connected. Normally, the sensor voltage wire would be wired to a relay. With the Hypnos, the power wire will be connected to a voltage rail on the Hypnos which are rated 3.3 V, 5 V, or the external voltage rail which is noted in Fig. 20. Connecting these sensor wires to the corresponding pins on the Feather would give the appropriate power, but would not have the power management functionality as connecting them to the voltage rails would.

**Fig. 21:**
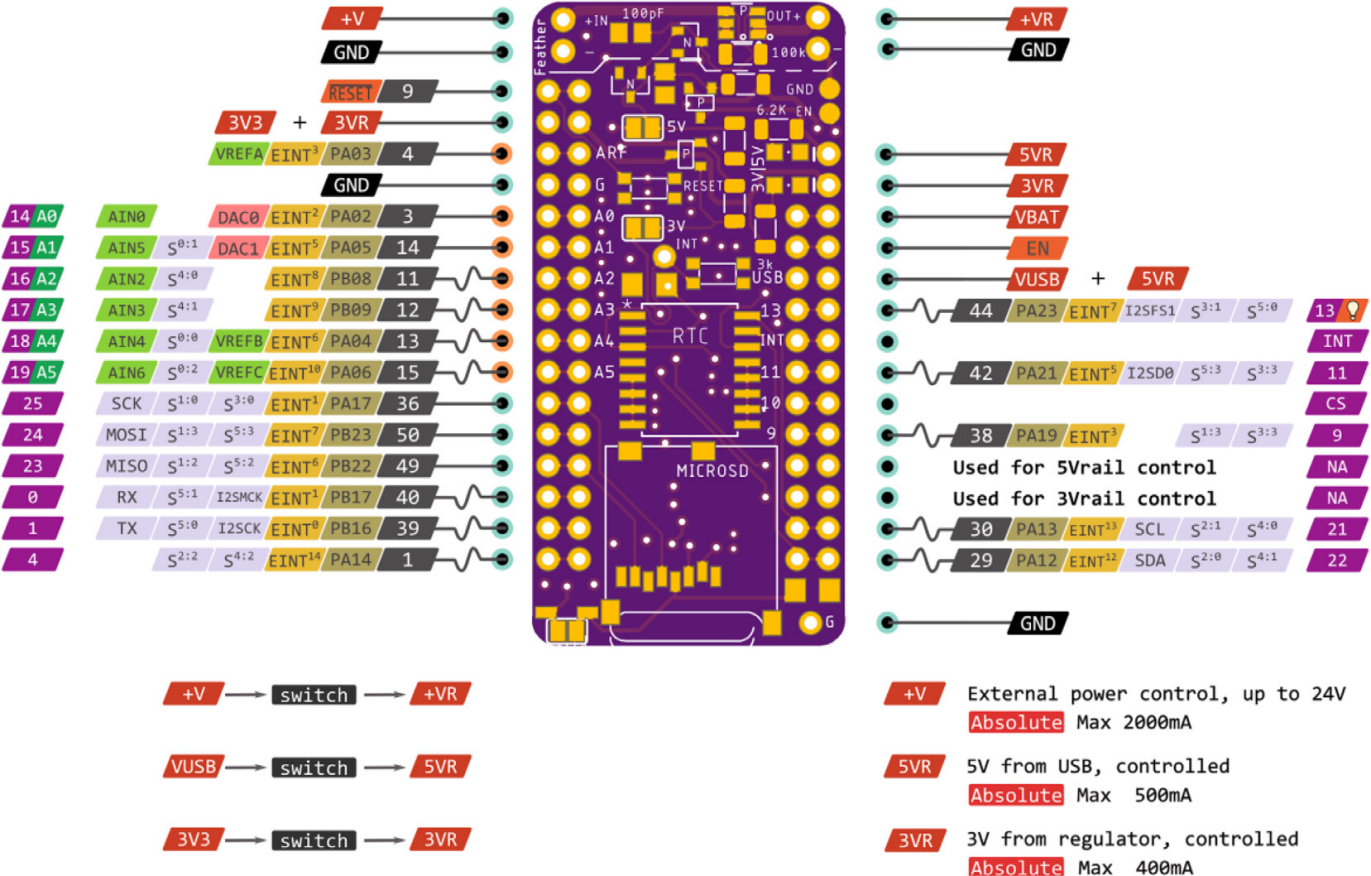
Hypnos Board Pinout Diagram.

The remaining wires will be connected to the pins they would normally be connected to the Feather. There are some exceptions to this rule-- meaning there are some pins that the user will not be able to use to wire their sensor which are pins 5 and 6 on the Hypnos. These pins are reserved for controlling the power rails. There are other pins that are reserved for Hypnos functionality such as pins 10 and 12 which are the chip select and interrupt pins respectively. These are noted in the pin diagram in Fig. 21 below.

### If a + V rail is necessary for the circuit, attach power source to the + V input

**Fig. 22:**
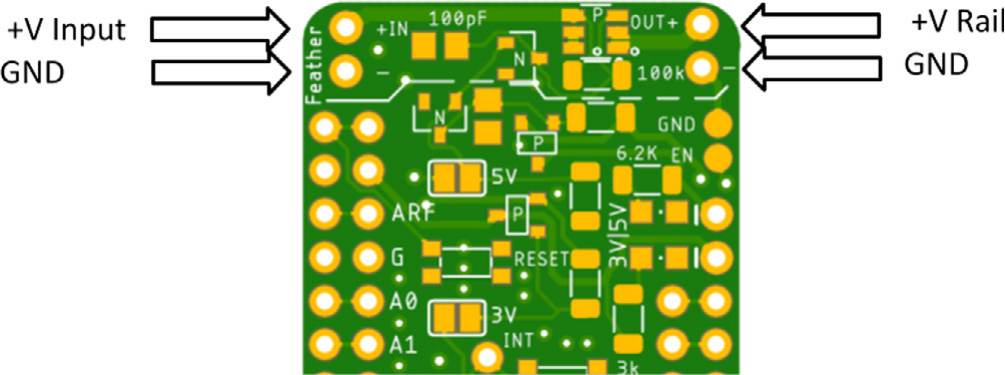
+ V rail Pins.

The external battery source can be connected to have a voltage output of the same rating with the added benefit of the power relay. The battery source would be connected to the + V Input and GND pins on the left side listed in Fig. 22 below, and the sensor wires would be connected to the + V rail and GND pins on the right side. This step is only necessary if the circuit requires a voltage level that is not rated at 3.3 V or 5 V. The maximum load that this voltage rail can tolerate is 24 V-- limited by the large P-channel MOSFET.

## Operation instructions

### Programming

#### Loom

The OPEnS lab uses an open source Arduino library called Loom which enhances the Hypnos programming process. By using this library, programming and prototyping with the Hypnos becomes much easier because most of the functionality of time keeping, data collecting, and data logging is done automatically. The repository for this library can be found in the OPEnS Loom Zenodo page and the example code within Loom to use the Hypnos can be found in Examples > Lab Examples > Hypnos_SD or Hypnos_SD_Sleep in the Loom Zenodo repository.

In Loom, there are built in functions that make programming easier. One aspect is the configuration json file that will read which sensors are being used. By including the SD and RTC in this json file, the program will recognize these components in the circuit. Once the system knows the components, there are other functions that record data from the sensors as well as log them to the μSD.

The program will be uploaded to the Feather M0 and the Hypnos will be attached through headers. The program will turn on the 3.3 V and 5 V power rails, initialize the RTC and SD, and take measurements taken by the Feather (including the time from the RTC and any sensor measurements) and log it to the SD.

#### Non Loom

The following code is used to operate the Hypnos’ SD, RTC, and power rail functionality without Loom. The RTC functionality was forked from Garrysblog’s GitHub repository and the SD functionality was based on Adafruit’s SD example. The values read from an analog pin are also read and written to a text file on the SD card. Note that the “SD.h” and “RTClib.h” libraries are necessary to compile the code. Installing Arduino should include the “SD.h” library, but the “RTClib.h” needs to be downloaded from Adafruit’s GitHub repository. Instructions on installing libraries can be found on Arduino’s Installing Additional Arduino Libraries page.

**Table:** 

#include < SD.h>
// Date and time functions using a DS3231 RTC connected via i2c and Wire lib
#include “RTClib.h”
RTC_DS3231 rtc;
const int alarmPin = 12;
const int sdPin = 10;
const int analogPin = A0; // Analog pin to take values from
File myFile; // Test file object to write to on SD
void setup()
{
Serial.begin(9600);
pinMode(5, OUTPUT);
digitalWrite(5, LOW); // Sets pin 5, the pin with the 3.3 V rail, to output and enables the rail
pinMode(6, OUTPUT);
digitalWrite(6, HIGH); // Sets pin 6, the pin with the 5 V rail, to output and enables the rail
pinMode(sdPin, OUTPUT);
pinMode(alarmPin, INPUT_PULLUP);
// Checking if SD and RTC is active
if (!SD.begin(sdPin)) {
Serial.println(“initialization failed!”);
return;
}
Serial.println(“initialization done.”);
if (! rtc.begin()) {
Serial.println(“Couldn't find RTC”);
Serial.flush();
abort();
}
if (rtc.lostPower()) {
Serial.println(“RTC lost power, let's set the time!”);
// When time needs to be set on a new device, or after a power loss, the
// following line sets the RTC to the date & time this sketch was compiled
rtc.adjust(DateTime(F(__DATE__), F(__TIME__)));
// This line sets the RTC with an explicit date & time, for example to set
// January 21, 2014 at 3am you would call:
// rtc.adjust(DateTime(2014, 1, 21, 3, 0, 0));
}
// Disables and clears alarms
rtc.disableAlarm(1);
rtc.disableAlarm(2);
rtc.clearAlarm(1);
rtc.clearAlarm(2);
DateTime now = rtc.now(); // Get current time
// Print current time and date
char buff[] = “Start time is hh:mm:ss DDD, DD MMM YYYY”;
Serial.println(now.toString(buff));
// Set alarm time
rtc.setAlarm1(now + TimeSpan(0, 0, 0, 10), DS3231_A1_Second); // In 10 s time
// open the file. note that only one file can be open at a time,
// so you have to close this one before opening another.
myFile = SD.open(“test.csv”, FILE_WRITE);
int testValue = analogRead(analogPin);
// if the file opened okay, write to it:
if (myFile) {
Serial.print(“Writing to test.csv…”);
myFile.println(testValue);
// close the file:
myFile.close();
Serial.println(“done.”);
} else {
// if the file didn't open, print an error:
Serial.println(“error opening test.csv”);
}
}
void loop()
{
if (rtc.alarmFired(1) == true){
// Print current time and date
DateTime now = rtc.now(); // Get the current time
char buff[] = “Alarm triggered at hh:mm:ss DDD, DD MMM YYYY”;
Serial.println(now.toString(buff));
// Disable and clear alarm
//rtc.disableAlarm(1); // Not used as only disables the alarm on the SQW pin
rtc.clearAlarm(1);
// Perhaps reset to new time if required
rtc.setAlarm1(now + TimeSpan(0, 0, 0, 10), DS3231_A1_Second); // Set for another 10 s
}
digitalWrite(5, HIGH); // Disabling all pins before going to sleep.
digitalWrite(6, LOW);
pinMode(23, INPUT); // Disables SPI communication to SD before going to sleep
pinMode(24, INPUT);
pinMode(10, INPUT);
delay(3000);
digitalWrite(5, LOW); // Enabling all pins after wake up has completed.
digitalWrite(6, HIGH);
pinMode(10, OUTPUT); // Enables SPI communication to SD after going to sleep
pinMode(23, OUTPUT);
pinMode(24, OUTPUT);
}

If the provided sketch does not work, there are several common issues that can be checked for:

• Is the appropriate board profile selected?
• Is the SD plugged in? Does it use FAT16 or FAT32 format?
• Are the necessary libraries correctly installed?

### Safety concerns

There are some potential safety issues with using the Hypnos board. Just like with any circuit, be sure to double check that the wiring from the sensors to the Hypnos board is correct. One important thing to keep in mind is to connect the external battery source correctly on the + V rail. Reversing this could potentially damage both the sensor as well as the Feather. Using the + V rail gives the user a lot of options to work with, however, there is a limit to the + V rail. To be safe, the highest voltage that should be attached to the + V rail is 24 V. The MOSFET is not designed to handle higher voltages so hooking up a higher voltage source may damage the circuit.

## Validation and characterization

**Fig. 23:**
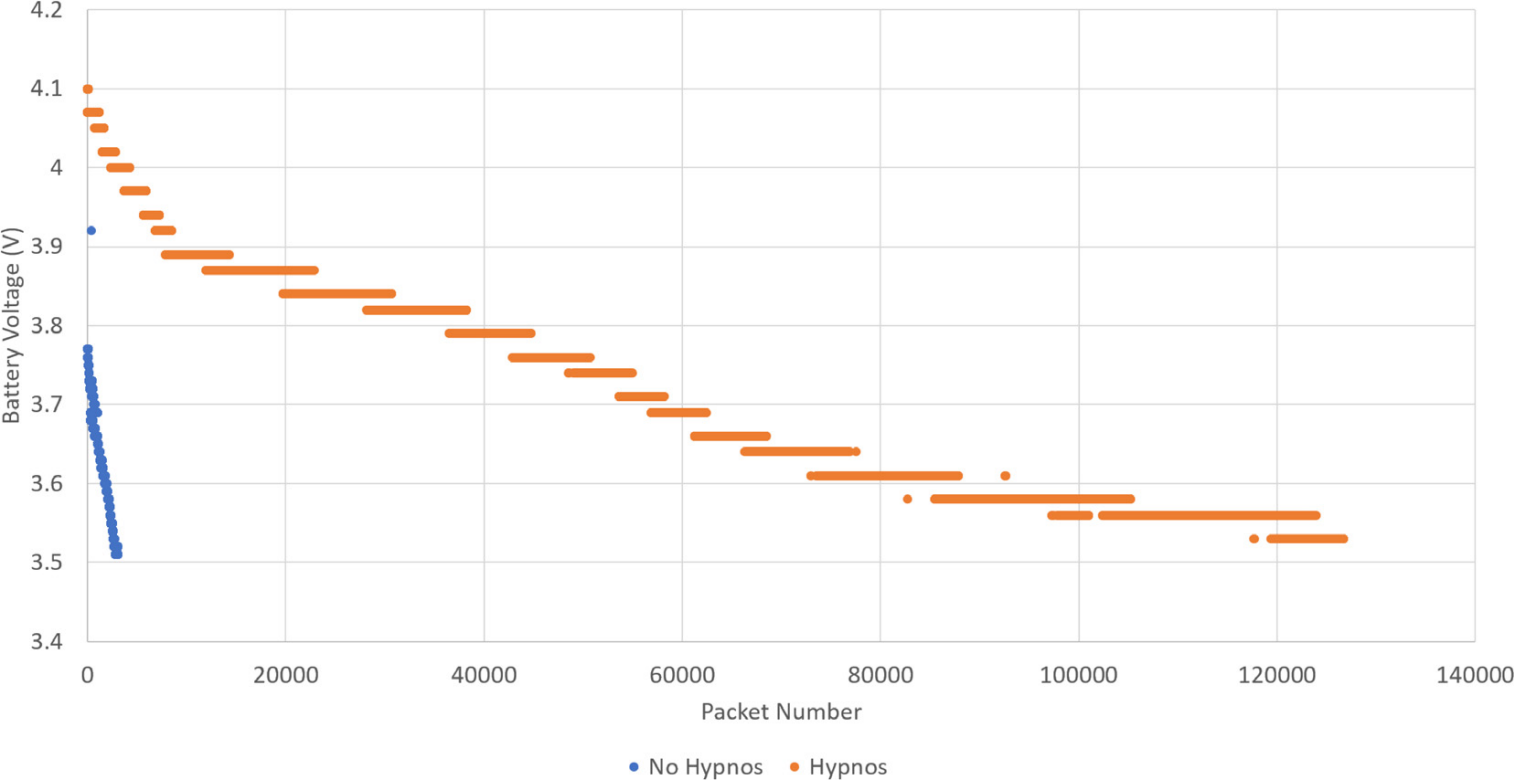
Battery Performance of SitkaNet.

**Fig. 24:**
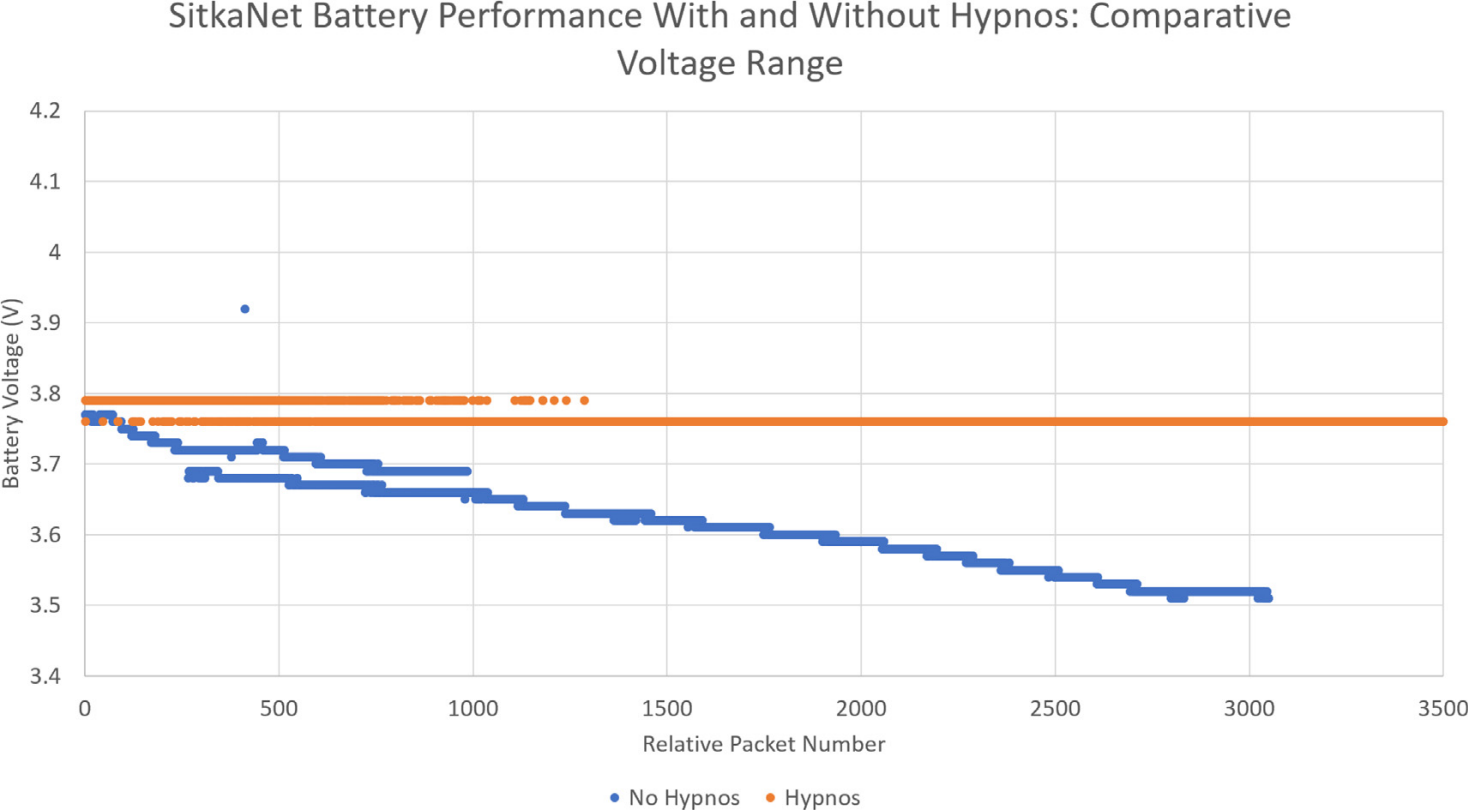
Focused Battery Performance of SitkaNet.

Data was taken from a project, Sitkanet, which has used the Hypnos board [14]. Two long term tests have been done, one with and one without the Hypnos board. During the testing, the battery levels of both systems were charted during their deployments. A 3.7 V, 6600mAh LiPo battery was connected to the Feather. In Fig. 23, the battery level of the entire deployment is shown while Fig. 24 shows the battery levels with relative battery levels for a fair comparison. Note that the battery level for the trial without the Hypnos board started at a lower value than the battery level with the Hypnos.

The duration of the deployment over these tests were different, which can be seen through the difference in packets charted. However, the data can be compared through relative voltage levels. There was a 5 min interval in between samples for both tests.

For a comparative range of voltages (3.76/3.77 V to 3.53/3.51 V), the system using the Hypnos took 82,282 samples while the system without the Hypnos took 3,050 samples-- meaning the trial with the Hypnos took over 25x more samples with the same amount of battery usage. This can be seen in the chart from the much steeper slope for the No Hypnos data. With the reduced sleep current (current between sample times), much less power is wasted and the battery levels last longer as shown in Table 1.

**Table 1:** Battery Level Comparison Table.

	Hypnos	No Hypnos	
Starting Comparative Voltage	3.76	3.77	V
Ending Comparative Voltage	3.53	3.51	V
Comparative Voltage Drop	0.23	0.26	V
Comparative Samples	83,282	3,050	
Average Voltage Drop / Sample Cycle	2.8	85	μV
Full Battery Voltage	4.1		V
Ending Battery Voltage	3.53		V
Total Voltage Drop	0.57		V
Total Samples	126,705		
Average Voltage Drop / Sample Cycle over entire Hypnos battery life	4.50		μV

## Human and animal rights

The work does not use any human or animal subjects.

## Declaration of Competing Interest

The authors declare that they have no known competing financial interests or personal relationships that could have appeared to influence the work reported in this paper.

## References

[1] Waheed M., Ahmad R., Ahmed W., Drieberg M., Alam M.M. (2018). Towards efficient wireless body area network using two-way relay cooperation. Sensors.

[2] P.A. Beddows E.K. Mallon 18 2 530 10.3390/s18020530

[3] Trevathan J., Johnstone R. (2018). Smart environmental monitoring and assessment technologies (SEMAT)—a new paradigm for low-cost. Remote Aquat. Environ. Monit. Sens..

[4] González A., Olazagoitia J.L., Vinolas J. (2018). A low-cost data acquisition system for automobile dynamics applications. Sensors.

[5] L. Ada, “Introducing Adafruit Feather,” Adafruit Learning System, 14-May-2017. [Online]. Available: https://learn.adafruit.com/adafruit-feather/feather-specification. [Accessed: 09-May-2021].

[6] NXP Semiconductors, “PCF8523,” PCF8523 v.7, Apr 28, 2015.

[7] Maxim Integrated, “DS3231,” DS3231 v.10, Mar 19, 2015.

[8] L. Ada, “Adafruit Feather M0 Basic Proto,” Adafruit Learning System. [Online]. Available: https://learn.adafruit.com/adafruit-feather-m0-basic-proto/pinouts. [Accessed: 03-May-2021].

[9] M. Marwell, “Issues with the I2C (Inter-IC) Bus and How to Solve Them,” DigiKey, 09-Aug-2018. [Online]. Available: https://www.digikey.com/en/articles/issues-with-the-i2c-bus-and-how-to-solve-them. [Accessed: 03-May-2021].

[10] Arduino Tutorials, “Tutorial on Arduino Watchdog Timer,” Circuits4you.com, 24-Jan-2018. [Online]. Available: https://circuits4you.com/2018/01/24/tutorial-on-arduino-watchdog-timer-setup/. [Accessed: 17-Jun-2021].

[11] Koontz, N (2020) Feather Fault (v1.1.7) [Source code]. https://github.com/OPEnSLab-OSU/FeatherFault.

[12] Pad2Pad, “Free Online Gerber Viewer: Circuit Board Rendering,” GerbLook. [Online]. Available: https://www.gerblook.org/. [Accessed: 09-May-2021].

[13] Solder Paste in cartridge 500g (T3) Sn42/Bi57.6/Ag0.4 Low Temperature. [Online]. Available: https://www.chipquik.com/store/product_info.php?products_id=540006. [Accessed: 24-Nov-2020].

[14] Chu M., Patton A., Roering J., Siebert C., Selker J., Walter C., Udell C. (2021). SitkaNet: A low-cost, distributed sensor network for landslide monitoring and study. HardwareX.

